# Reactive Fluid Ferroelectrics: A Gateway to the Next Generation of Ferroelectric Liquid Crystalline Polymer Networks

**DOI:** 10.1002/smll.202501724

**Published:** 2025-04-07

**Authors:** Stuart R Berrow, Jordan Hobbs, Calum J Gibb

**Affiliations:** ^1^ Department of Physics and Astronomy University of Leeds Leeds LS2 9JT UK; ^2^ Department of Chemistry University of Leeds Leeds LS2 9JT UK

**Keywords:** ferroelectric materials, liquid crystals, materials science, reactive mesogens

## Abstract

Herein it is reported the first examples of reactive mesogenic materials (RMs) which exhibit fluid ferroelectric order based on the recently discovered ferroelectric nematic (N_F_) phase. These materials N_F_ RMs and they provide the first steps toward the next generation of ferroelectric liquid crystalline polymer networks is termed. The chemical synthesis and characterization of the liquid crystalline properties of these materials is reported, demonstrating that they have the lowest longitudinal molecular dipole moments (µ) of any reported N_F_ material of 7.39 D. It is go on to demonstrate a potential use case of this new class of reactive material through the polymer stabilization of a matrix which exhibits the N_F_ phase, increasing the phase range of the ferroelectric phase from 75 to 120 °C. The N_F_ RMs reported herein are an exciting step forward in ferroelectric liquid crystal research, demonstrating that reactive N_F_ materials are achievable, allowing for the future development of liquid crystalline ferroelectric networks, elastomers and polymers.

## Graphical abstract

The first examples of reactive mesogenic materials (RMs) which exhibit fluid ferroelectric order based on the recently discovered ferroelectric nematic (N_F_) phase is reported. It is termed these materials N_F_ RMs and they provide the first steps toward the next generation of ferroelectric liquid crystalline polymer networks.

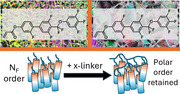

## 1 Introduction

Reactive mesogens (RMs) are functional materials displaying liquid crystalline (LC) mesophases that possess reactive groups, allowing for the formation of polymers, elastomers and network structures with liquid crystalline order. RMs have existed for nearly 50 years, being developed as early interest into liquid crystal polymers emerged.^[^ ^1^, ^2^, ^3^, ^4^ ^]^ The scope of RMs began to grow in the late 1980s, when their potential applications were explored. These began with investigations into their potential to produce functional coatings, for example for the protection of optical fibres,^[^ ^5^ ^]^ but it was not long until applications in the fabrication of liquid crystal displays (LCDs) emerged, leading to far reaching implications in the multibillion‐dollar LCD industry.^[^ ^6^, ^7^, ^8^, ^9^ ^]^ The applications for reactive mesogenic materials have continued to grow giving rise to new fields of study, such as: liquid crystal elastomers (LCEs),^[^ ^10^, ^11^ ^]^ liquid crystal templating,^[^ ^12^ ^]^ polymer dispersed LC networks,^[^ ^13^ ^]^ and the stabilisation of LCs for device applications – commonly referred to as polymer stabilised liquid crystals (PSLCs).^[^ ^14^ ^]^

The recent discovery of the ferroelectric nematic (N_F_) phase, which combines nematic orientational order and fluidity with almost perfect parallel polar order of its constituent molecules,^[^ ^15^, ^16^, ^17^, ^18^, ^19^ ^]^has garnered significant scientific interest due to its wide‐ranging potential applications.^[^ ^20^, ^21^, ^22^, ^23^, ^24^, ^25^ ^]^ The scope of the N_F_ phase would be further increased through its incorporation into network structures. While some work has already been done on macromolecular N_F_ materials,^[^ ^26^, ^27^, ^28^ ^]^ a polymer network with inherent N_F_ order has yet to be reported but would be desirable.^[^ ^29^ ^]^ In order to attain a network with inherent N_F_ ordering, an RM exhibiting the N_F_ phase would be ideal, as it could facilitate pathways to networks that are polar in their own right. To date, there is no such RM reported within the literature and so we elected to design and synthesise a ferroelectric nematogen with reactive functionality.

## 2 Results and Discussion

### 2.1 Monomer Synthesis and Properties

As research into the N_F_ phase is still largely in its infancy, there are only a few chemical structure spaces currently known to promote the formation of the N_F_ phase.^[^ ^30^, ^31^, ^32^ ^]^ 4‐(difluoro(3,4,5‐trifluorophenoxy)methyl)‐1,1′‐biphenyl) is a common molecular moiety present in several materials exhibiting the N_F_^[^ ^33^, ^34^ ^]^ (and other polar)^[^ ^35^, ^36^, ^37^ ^]^ mesophases. As such, we elected to choose this as the basic unit for our RMs, coupling variations of the biphenyl unit with different fluorine substitution patterns to simple acrylate units to afford the four reactive materials **1–4**. The transitional properties of **1–4** were initially examined by differential scanning calorimetry (DSC) and polarised optical microscopy (POM) affording the phase behaviour outlined in (**Table** **1**).

**Table 1:** Transition temperatures and their associated enthalpy changes [in brackets] for **1–4**. Their longitudinal molecular dipole moments (µ / D) (at the DFT:B3LYP‐GD3BJ/cc‐pVTZ level of theory^[^ ^41^, ^42^, ^43^, ^44^, ^45^ ^]^) are also given in the left‐hand column. Crystallization temperatures (Crystal) recorded on cooling.

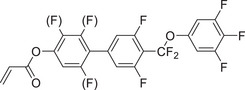				
Compound	Melt	SmA_F_ – N_F_	N_F_‐I	N‐I
	T / °C [*Δ*H / KJ mol^−1^]	T / °C [*Δ*H / KJ mol^−1^]	T / °C [*Δ*H / KJ mol^−1^]	T / °C [*Δ*H / KJ mol^−1^]
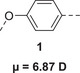	73.9 [25.8]	–	–	77.1 [0.5]
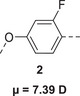 **2**	81.6 [15.2]	27.7 [0.5]	84.3 [2.9]	–
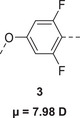	112.4 [21.6]	–	116.2 [5.7]	–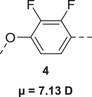	103.0 [32.4]	–	–	–

**Figure 1:**
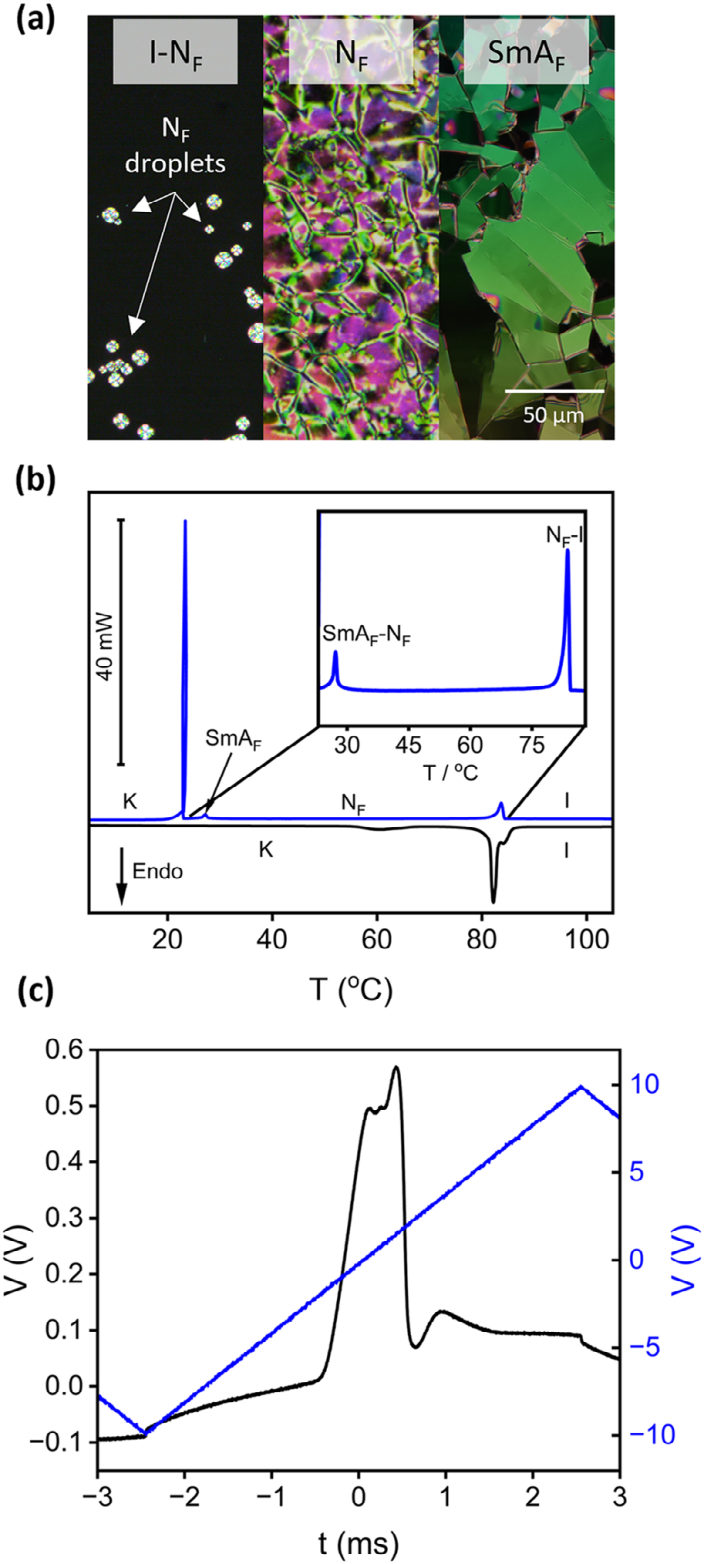
a) POM micrographs taken for **2** depicting **[left]** the I‐N_F_ phase transition where the N_F_ phase forms from droplets, **[centre]** the banded texture of the N_F_ phase, and **[left]** the blocky mosaic structures associated with the SmA_F_ phase. Images were taken within thin cells with no alignment layer at 84, 50, and 25 °C, respectively; b) DSC thermogram of **2** showing heating and cooling traces; and (c) current response trace measured for **2** measured at 100 Hz in the N_F_ phase at 80 °C.

Of the four RMs prepared, **1** shows simple apolar nematic behaviour, **2** and **3** exhibit enantiotropic N_F_ phases, and **4** is non‐mesogenic. When viewed under POM between untreated glass, the conventional N phase of **1** was assigned by the observation of a Schlieren texture containing 2‐ and 4‐ point brush singularities which flash under mechanical stress (Figure S6a, Supporting Information) – characteristic of the apolar N phase. For both **2** and **3**, cooling from the isotropic (I) phase sees the N_F_ phase form from small droplets (**Figure** **1**a **[left]**), characteristic of the N_F_ phase.^[^ ^38^ ^]^ Further cooling sees the coalescence of the droplets into a banded texture, synonymous with the assignment of the N_F_ phase (Figure 1a **[centre]**). For **2**, a good degree of super‐cooling can be achieved the N_F_ phase being observed for over 60 °C before a further transition result in the appearance of a blocky mosaic texture (Figure 1a **[right]**) which is commonly associated with the SmA_F_ phase.^[^ ^39^, ^40^ ^]^ Attempts to confirm this assignment via X‐ray scattering were unsuccessful due to crystallisation of the sample during measurements and, therefore, the assignment of the SmA_F_ phase here is tentative. **3** shows significantly less super‐cooling and so only exhibits the N_F_ phase from 116—100 °C. Ferroelectric order within the N_F_ phases was confirmed via current response measurements (Figure 1c; Figure S7, Supporting Information), where a single polarisation– reversal peak was observed, along with a second smaller peak associated with ion flow.

Due to the relatively recent discovery of the N_F_ phase, the molecular origins of the N_F_ phase are still emerging, and it is therefore difficult to rationalise the drastically different phase behaviour of **1–4** despite their very similar chemical structures. Initially, the N_F_ phase was observed in molecules with very large longitudinal molecular dipole moments (µ) (circa 11 D) and so it was suggested that this was a pre‐requisite for the formation of the phase.^[^ ^24^, ^46^ ^]^ Subsequent studies have revealed examples of materials exhibiting polar mesophases, particularly smectic materials, with comparatively smaller dipole moments (circa 7–8 D)^[^ ^47^, ^48^, ^49^ ^]^ indicating that the either the dipole limit is much lower than initially suspected or that the absolute value of µ is not be sole determinant factor in the formation of polar LC phases. It has been shown that for dipolar spheres the global minima is not parallel orientation of neighbouring molecules^[^ ^50^, ^51^ ^]^ and so it seems there must be some further contribution to the molecular origin of polar order. Notably, **1–4** all possess comparatively low values of µ (Table 1) yet **2** and **3** both exhibit the N_F_ phase. Of particular interest is **2** which possesses–the lowest reported dipole moment of any ferroelectric nematogen at 7.39 D at the DFT:B3LYP‐GD3BJ/cc‐pVTZ level of theory.^[^ ^41^, ^42^, ^43^, ^44^, ^45^ ^]^

**Figure 2:**
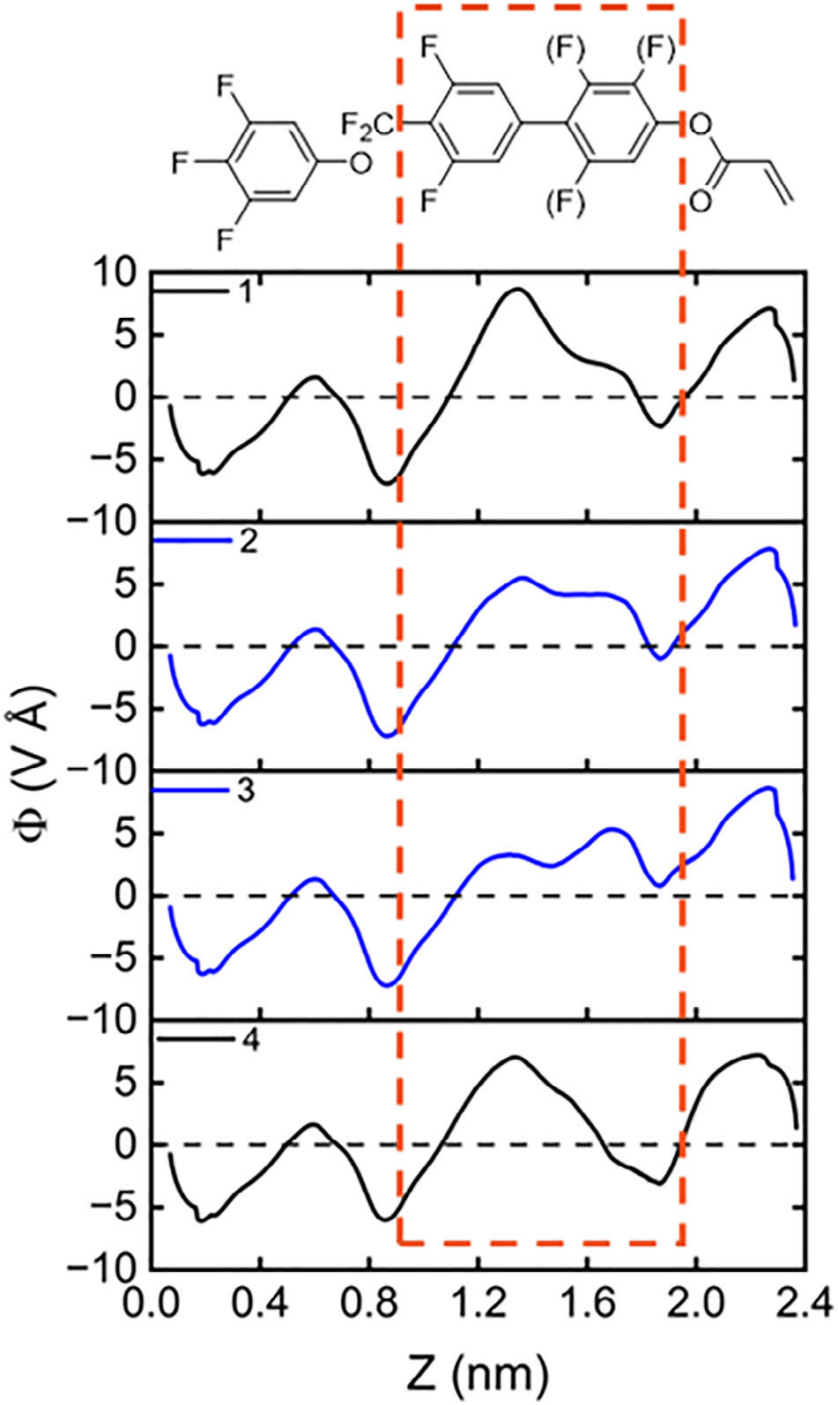
1D longitudinal charge density wave calculated for 1–4 (B3LYP‐GD3BJ/cc‐pVTZ level of theory).^[^ ^41^, ^42^, ^43^, ^44^, ^45^ ^]^ The more regular oscillatory structure of the charge density wave for **2** and **3** implies more even spatial distribution of the 3D ESP surface (Figures S11–S14, Supporting Information) leading to the observation of the N_F_ phase.

Madhusudana has suggested that specific oscillating charge distributions along the long molecular axis can provide a pathway for molecules to adopt parallel orientations (i.e., broken inversion symmetry).^[^ ^52^ ^]^ This mitigates the propensity for adopting antiparallel conformations present in the conventional, apolar N phase.^[^ ^53^, ^54^ ^]^ This model has already been applied and shown to work for archetypal ferroelectric nematogens such as RM734^[^ ^55^, ^56^ ^]^ and DIO,^[^ ^57^, ^58^ ^]^ and it is now being refined and applied to other structure spaces using computational DFT studies to visualise the ESP^[^ ^47^, ^49^ ^]^ where it has been suggested that an additional component to this model is good 3D spatial uniformity of the charge density.^[^ ^49^ ^]^ By applying a similar computationally driven approach to **1–4** (details given in the ESI) it is possible to visualise the ESP as a function of molecular length (**Figure** **2**). For **2** and **3** which exhibit the N_F_ phase, charge density oscillates almost sinusoidally across the biphenyl structure (highlighted within the orange box in Figure 2) with small changes in the amplitude of the oscillations. For **1** and **4**, the amplitude of these oscillations are much more pronounced, lacking the clear oscillatory structure observed for **2** and **3**. These observations are consistent with previous studies^[^ ^47^, ^49^, ^59^ ^]^ and, at least empirically, reinforces the emerging observation that sinusoidal structure in the 1D ESP distribution is a good charge structure for the observation of the N_F_ phase.

### 2.2 Polymer Stabilization Investigations

An obvious direction for the application of N_F_ RMs is in the polymer stabilisation of the N_F_ phase. While the N_F_ phase has huge application potential, current materials are limited by factors such as narrow temperature ranges, chemical stability and the propensity of these materials to crystallise at low temperatures. We envision that, as was the case with the conventional N phase and other mesophases,^[^ ^14^ ^]^ polymer stabilisation of the N_F_ phase would be a useful tool to overcome these shortcomings and increase the efficacy of materials toward device applications.

Current work into the stabilisation of the N_F_ phase have reported poor miscibility with network structures, typically resulting in the phase separation of polymerizable and non‐polymerizable components.^[^ ^60^ ^]^ These networks often consist of entirely non‐mesogenic materials,^[^ ^61^, ^62^ ^]^ or a combination of non‐mesogenic materials and symmetrical RMs with very low dipole moments.^[^ ^62^ ^]^ In the case of the RMs studied previously, they are symmetrical along their long molecular axis, thereby possessing negligible longitudinal dipole moments. It can therefore easily be envisioned that their negligible polarity would result in unfavourable interactions with highly polar materials like ferroelectric nematogens. Previous reports do not provide a rationale for the observed poor miscibility. However, this is likely a result of the poor mixing of polar and apolar mesophases.^[^ ^63^ ^]^ Further to this, it has also been shown that large quantities of non‐mesogenic components can lead to phase separation, likely resulting from separation of alkyl chains and aromatic units.^[^ ^64^ ^]^ While some successes have been achieved using blue‐phase networks,^[^ ^60^ ^]^ and optically isotropic polymer stabilised materials,^[^ ^61^, ^62^ ^]^ presently, polymer stabilisation of the N_F_ phase has not yet been reported. We envisaged that if the network contains repeat units that exhibit the N_F_ phase, there will be two advantages. First, the issue of poor affinity will be mitigated by the ideal mixing behaviour that occurs between N_F_ molecules in mixtures.^[^ ^65^ ^]^ Second, it is likely that a key component for the formation of a polar network is to polymerise in a polar phase. The addition of a non‐N_F_ RM would cause a significant reduction in the N_F_ transition temperature^[^ ^63^ ^]^ preventing polymerisation in the N_F_ phase. By using an N_F_ RM, this is avoided completely.

**Figure 3:**
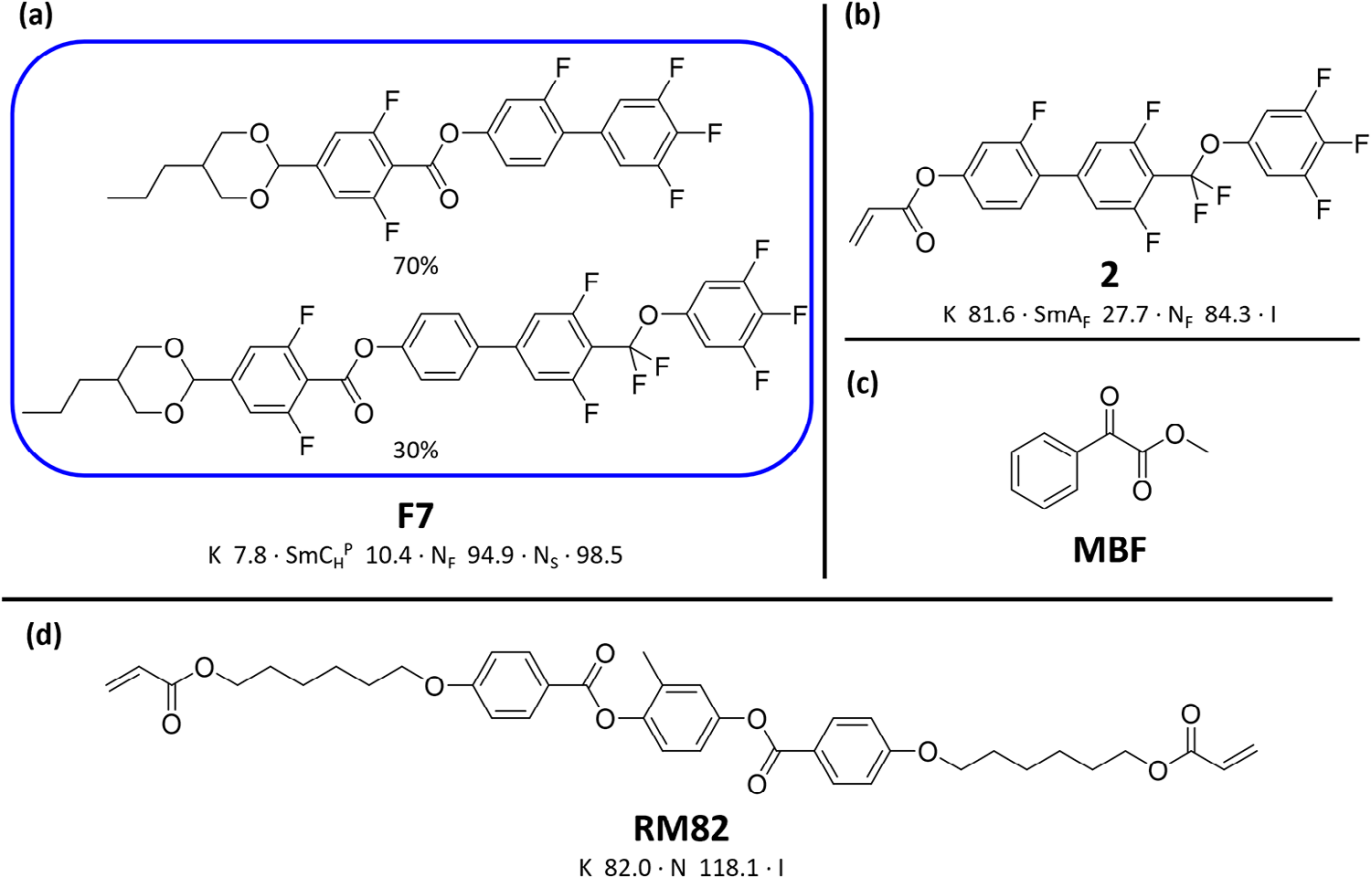
Chemical structures and their transition temperatures, where appropriate, of a) the room temperature ferroelectric nematic mixture **F7**, b) the N_F_ RM 2 (presented in this work), c) the photoinitaitor methyl benzoylformate (**MBF**), and d) the crosslinker **RM82** used in the formulation of mixtures **PS1‐4**.

**Figure 4:**
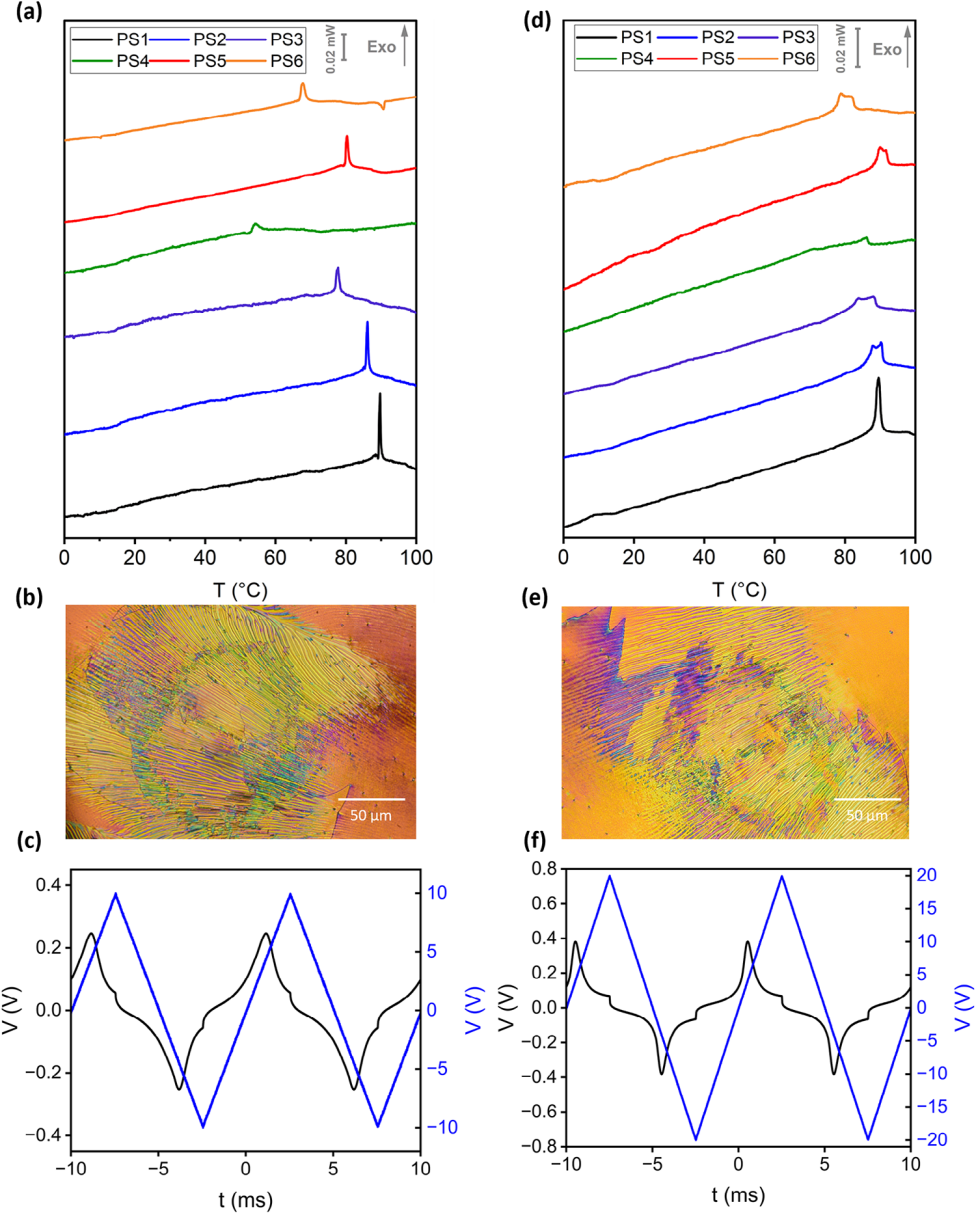
a) DSC thermograms of PS1‐4, b) POM micrograph of PS2 at 40 °C, and c) current response trace for PS2 measured at 100 Hz in the N_F_ phase at 30 °C pre‐polymerisation. d) DSC thermograms of PS1‐4, e) POM micrograph of PS3, and f) current response trace for PS3 measured at 100 Hz in the N_F_ phase at 30 °C post‐polymerisation.

For our test, we elected to stabilise the phase behaviour of the simple, unreactive mixture **F7** (**Figure** **3**a),^[^ ^35^ ^]^ a simple binary mixture of two polar materials which exhibits the N_F_ phase from room temperature up to 95 °C before transitioning to the splay nematic (N_S_) phase (an antiferroelectric nematic phase sometimes referred to as N_X_, N_AF_ or SmZ_A_).^[^ ^66^, ^67^ ^]^ Notably, both the components of **F7** contain a 2,5‐disubstituted 1,3‐dioxan moieties which is known to isomerise at high temperatures, resulting in a decrease in transition temperatures.^[^ ^47^ ^]^ As such, mixtures containing **F7** are only heated to a maximum temperature of 120 °C as this mitigates any changes in transition temperatures due to isomerisation (Figure S11, Supporting Information). A series of mixtures containing varying quantities of **2** (Figure 3b), the photoinitiator **MBF** (Figure 3c), and the commercially available crosslinker **RM82** (Figure 3d) in **F7** were produced, ensuring that in the first instance, the concentrations of **F7** and **MBF** were kept constant (**Table** **2** **; PS1‐PS4**). Prior to polymerisation, each mixture was examined using the same physical characterisation techniques as materials **1–4**, confirming that the N_F_ phase was exhibited by each mixture (**Figure** **4**a–c; Figures S8 and S9, Supporting Information). For **PS3** and **PS4** the transition temperatures associated with the N_F_ phase fall dramatically compared to **F7** due to the increased quantity of apolar **RM82** expectedly destabilising polar order.

**Table 2:** The compositions of the polymer stabilised mixtures **PS1‐4**, and the phase transition temperatures for the crystal to ferroelectric nematic (K‐N_F_) and ferroelectric nematic to anti‐ferroelectric nematic (N_F_‐N_s_) phase transitions pre‐ and post‐polymerisation.

Mixture Name	2 [wt.%]	RM82 [wt.%]	MBF[wt.%]	F7[wt.%]	Pre‐polymerization		Post‐Polymerization	
					T_K‐NF_ (°C)	T_NF‐NS_ (°C)	T_K‐NF_ (°C)	T_NF – NS_ (°C)
**PS1**	8.65	1.25	0.1	90	–	89.9	–	89.8
**PS2**	7.4	2.5	0.1	90	–	86.6	–	90.6
**PS3**	4.9	5.0	0.1	90	24.1	78.5	–	86.3
**PS4**	0	9.9	0.1	90	22.5	54.5	20.7	86.4
**PS5**	9.9	5.0	0.1	85	22.2	81.1	–	89.7
**PS6**	2.4	5.0	0.1	92.5	17.7	68.9	13.2	82.9

Post‐polymerisation using UV light (365 nm (2.5 W cm^−2^, 20 min), the N_F_ phase is retained for all four mixtures and an increase in the temperature stability of the N_F_ phase is observed for **PS2‐4** with the transition temperature of the N_F_ phase in **PS1** remaining comparable to the uncured sample (Table 2, Figure 4d). This is due to the low concentration of **RM82** present in this mixture indicating a minimum degree of crosslinking is required for stabilisation. The characteristic banded texture of the N_F_ is retained post‐polymerisation by all mixtures (Figure 4e) indicating that this polymer stabilisation mixture does not prevent the separation of the N_F_ texture into domains of alternative polarisation. A stronger electric field is required to facilitate the switching of the polymerized materials (Figure 4f) likely due to an increased elastic deformation cost which could be due to the creation of stronger polar anchoring by the now polymerised network or some other restriction imposed by the resulting network structures. There is also likely to be an element of increased rotational viscosity.

The largest degree of stabilisation in the N_F_ phase is seen for **PS4**, a mixture containing no **2** and, to a first approximation, it appears that an N_F_ RM is not required to successfully stabilise the N_F_. However, both a significant broadening of the first order peak associated with the N_F_ transition (Figure 4) and the cold crystallisation of the sample on re‐heating (Figure S8, Supporting Information) are evidence of significant phase separation due to the incompatibilities of mixing polar and apolar mesophases.^[^ ^63^ ^]^ Although the degree of stabilisation, as judged by the increase in T_NF‐NS_, in **PS2** and **PS3** is lower than that observed in **PS4**, there is clearly significantly less de‐mixing (Figure S8d, Supporting Information). While incorporating a N_F_ RM into the mixture reduces the effect of the stabilisation, its presence is clearly beneficial, enhancing the miscibility between the network and the matrix. This gives rise to wider working temperature ranges of the N_F_ phase, and therefore providing an overall benefit to the inclusion of **2**.

To further asses the parameters surrounding the stabilisation of the N_F_ phase, two further mixtures were produced (**PS5** and **PS6**). These mixtures contain the same quantity of cross‐linker as **PS3** (5 wt.%) but vary by way of the quantity of **2** present, thereby yielding networks of varying cross‐link density. Of these three mixtures, **PS6**, which has the greatest cross‐link density shows the largest degree of stabilisation, consistent with our findings for **PS1**‐**PS4**; However, while **PS5** has a lower cross‐link density than **PS3**, it shows a larger degree of stabilisation. This perhaps suggests that above a sufficient cross‐link density, the effect of enhancing network miscibility may outweigh that of increased cross‐link density.

These observations illustrate a complexity to polymer stabilisation of the N_F_ phase. While in general, stabilisation is greatest for networks with higher cross‐link density, we have demonstrated that the current commercially available cross‐linkers are prone to de‐mixing and phase separation when applied to N_F_ matrices. The incorporation of RMs with N_F_ character improves miscibility of the network and matrix, clearly benefiting the materials as a whole and in some cases appearing to enhance stabilisation. The challenge of optimising N_F_ phase polymer stabilisation is clearly complex and will be the subject of future studies. We also suggest that cross‐linkable N_F_ RMs would be desirable, to further the scope for development, which too will be the subject of future work.

## 3 Conclusion

To summarise, we have reported the synthesis of the first reactive mesogen materials to natively display the N_F_ phase which we term N_F_ RMs. We then show a potential use case for this new class of functional materials through the polymer stabilisation of the matrix **F7**.^[^ ^35^ ^]^ When mixed in conjunction with a small amount of crosslinker (**RM82**) and photoinitiator (**MBF**), we demonstrate the successful stabilisation of the N_F_ matrix increasing the effective N_F_ phase range from 75 to 120 °C. By using inherently ferroelectric RMs we act to mitigate the phase separation issues that have been problematic in the previously attempted network structures.^[^ ^28^ ^]^ We expect that N_F_ RMs may be pivotal in the production of new ferroelectric liquid crystalline networks, elastomers and polymers, thereby increasing the possible use cases of the N_F_ phase.

## Conflict of Interest

The authors declare no conflict of interest.

## Supporting information



Supporting Information

## Data Availability

The data that support the findings of this study are openly available in University of Leeds Data Repository at https://doi.org/10.5518/1635, reference number 1635.
