# Supplementary material for: Reactive Fluid Ferroelectrics: A Gateway to the Next Generation of Ferroelectric Liquid Crystalline Polymer Networks
Source: Small. 2025 Apr 7;21(21):2501724. doi: 10.1002/smll.202501724 (PMC12105451; doi:10.1002/smll.202501724)
Supplement: Supplementary file 1 — Supporting Information [file SMLL-21-2501724-s001.docx]

**Reactive Fluid Ferroelectrics: A Gateway to the Next Generation of Ferroelectric Liquid Crystalline Networks**

S. R. Berrow^1^, J. Hobbs^1^_,_ and C. J. Gibb^2*^

^1^School of Physics and Astronomy, University of Leeds, LS2 9JT

^2^School of Chemistry, University of Leeds, LS2 9JT

Correspondence: C.J.Gibb@leeds.ac.uk

**Electronic Supplementary Information**

**Supporting Information available**: Experimental information, synthetic procedures for the synthesis of all monomers and intermediates, NMR spectra, Characterisation of monomers (DSC, POM and current reversal) and polymer stabilisation studies (procedure, DSC and current reversal).

**Table Of Contents**

[Experimental Information 2](#_Toc192500288)

[Synthetic Procedures 4](#_Toc192500289)

[NMR Spectra 9](#_Toc192500290)

[Characterisation of Monomers 17](#_Toc192500291)

[Polymer Stabilisation Studies 19](#_Toc192500292)

[Three Dimensional Electrostatic Potential Computations 23](#_Toc192500293)

[References 25](#_Toc192500294)

## Experimental Information

### Materials

Chemicals were purchased from commercial suppliers (Fluorochem, Merck, ChemScene, Ambeed) and used as received. Solvents were purchased from Merck and used without further purification. Reactions were performed in standard laboratory glassware at ambient temperature and atmosphere (unless otherwise stated) and were monitored by TLC with an appropriate eluent and visualised with 254 nm light.

### Flash Chromatography

Flash chromatography was performed on a Combiflash NextGen 300+ system (Teledyne Isco) using silica as a stationary phase, an appropriate mobile phase as specified in the experimental procedure, and detection in the 200-800 nm wavelength range.

### Structural Analysis

Nuclear magnetic resonance (NMR) spectra were recorded using either a Bruker AVANCE III (400 MHz) NMR spectrometer (Bruker UK Ltd., Coventry, UK) or a Bruker AV4 NEO 11.75T (500 MHz) spectrometer, with spectra collected at 298 K and referenced to TMS. NMR spectra were viewed and analysed using MNova NMR software.

### Thermal Analysis

Differential scanning calorimetry (DSC) measurements were performed using a TA Instruments Q2000 DSC instrument (TA Instruments, Wilmslow UK), equipped with a RCS90 Refrigerated cooling system (TA Instruments, Wilmslow UK). The instrument was calibrated against an Indium standard, and data were processed using TA Instruments Universal Analysis Software. Samples were analysed under a nitrogen atmosphere, in hermetically sealed aluminium TZero crucibles (TA Instruments, Wilmslow, UK) and subjected to three analysis cycles. In all cases, samples were subject to heating and cooling at a rate of 10 K min^-1^. Phase transition temperatures were measured as onset values on cooling cycles for consistency between monotropic and enantiotropic phase transitions, while crystal melts were obtained as onset values on heating.

### Optical Microscopy

Polarised light optical microscopy (POM) was performed using a Leica DM2700P polarised light microscope (Leica Microsystems (UK) Ltd., Milton Keynes, UK), equipped with 10x and 50x magnification, and a rotatable stage. A Linkam TMS 92 heating stage (Linkam Scientific Instruments Ltd., Redhill, UK) was used for temperature control, and samples were studied sandwiched between two untreated glass coverslips. Images were recorded using a Nikon D3500 Digital Camera (Nikon UK Ltd., Surbiton, UK), using DigiCamControl software.

### Electronic Structure Calculations

Electronic structure calculations were performed using Gaussian G16 revision C.02 at a B3LYP-GD3BJ/cc-pVTZ [1-5] level of theory. Obtained structures were verified as a minimum from frequency calculations. Electrostatic potential (ESP) surfaces were calculated by using the *formchk* and *cubegen* utilities. Both the electron density and ESP cube files were calculated using “fine” data resolution. The 3D ESP surface is displayed at an electron density iso-surface of 0.0004.

The 3D data was reduced into 1D through the following steps. The electron density and ESP cube files are structured such that the long molecule axis of the molecule is centred along the z-axis of the data in each cube file. Each step in the z-axis is taken as a single plane through the molecule at that point. An iso-contour through the electron density cube file is found at some isovalue (here 0.0004 as used to mimic the 3D surfaces). The values of the ESP data that then fall on this iso-contour route are then found. These values reflect the 3D surface visualised in figure S6 exactly. We assume free rotation around the long molecule axis and so average the entire ESP data that falls along the iso-contour. This gives the average ESP value that a neighbouring molecule will “feel” for timescales longer that those of rotation around the long axis.

A further step of rescaling the values obtained by the length of the contour allows to account for the fact that at the molecular extremes the values are distorted by the reduction in molecular volume. This final step effectively gives the ESP as electric flux i.e. the strength of the electric field due to the molecular dipole through the contour.

### Measurement of Spontaneous Polarization (P_S_)

Spontaneous polarisation measurements are undertaken using the current reversal technique [6, 7]. Triangular waveform AC voltages are applied to the sample cells with an Agilent 33220A signal generator (Keysight Technologies), and the resulting current outflow is passed through a current-to-voltage amplifier and recorded on a RIGOL DHO4204 high-resolution oscilloscope (Telonic Instruments Ltd, UK). Heating and cooling of the samples during these measurements is achieved with an Instec HCS402 hot stage controlled to 10 mK stability by an Instec mK1000 temperature controller. The LC samples are held in 4µm thick cells with no alignment layer, supplied by Instec. The measurements consist of cooling the sample at a rate of 1 Kmin^-1^ and applying a set voltage at a frequency of 100 Hz. The voltage was set such that it would saturate the measured P_S_ and was determined before final data collection.

There are three contributions to the measured current trace: accumulation of charge in the cell (I_c_), ion flow (I_i_), and the current flow due to polarisation reversal (I_p_). To obtain a P_S_ value, we extract the latter, which manifests as one or multiple peaks in the current flow, and integrate as:

$P_{S}=\int\frac{I_{p}}{2A}dt$ **(1)**

where A is the active electrode area of the sample cell.

## Synthetic Procedures

The synthetic route to the four monomers is displayed in **Scheme S1**. The synthesis of intermediate **I1** and the four phenolic reagents (**I2-5**) are reported elsewhere [8-10].

**Scheme S1** – The synthetic pathway employed in the synthesis of monomers **1-4**.

### General Procedure for Synthesis of Monomers 1-4

A round bottomed flask was charged with the appropriate phenol (1 mmol, 1.0 eq), triethylamine (1.5 mmol, 0.21 mL, 1.5 eq) and THF (conc. ~ 0.2 M) and the flask cooled over an ice-water bath. To the reaction mixture was added acryloyl chloride (0.12 mL, 1.5 mmol) dropwise of a period of 15 mins, before the reaction mixture was allowed to warm to room temperature and stirred overnight. The solvent was removed under reduced pressure, and the resulting material dissolved in chloroform (100 mL). The organic layer was washed with aqueous NaHCO_3_ solution (1M) (2x100 mL) and brine (saturated) (100 mL), and the organic layer dried over magnesium sulphate. The solvent was removed under reduced pressure, and the crude solid recrystallized from isopropanol, to yield the products as colourless crystals.

*4-acryloxy-(3’,5’-difluoro-4’-(difluoro(3,4,5-trifluorophenoxy)methyl)-1,1’-biphenyl)* **(1)**

Yield: (Colourless crystals) 180 mg, 40%

R_F_ (50:50 Hexane:DCM): 0.47

^1^H NMR (501 MHz, CDCl_3_) (δ): 7.51 (ddd, J = 8.7, 2.0, 2.0 Hz, 2H, Ar-**H**), 7.19 (ddd, J = 8.7, 2.0, 2.0 Hz, 2H, Ar-**H**), 7.12 (d_apparent_, J = 10.2 Hz, 2H, Ar-**H**), 6.91 (dd, J = 7.9, 5.8 Hz, 2H, Ar-**H**), 6.57 (dd, J = 17.3, 1.2 Hz, 1H, HC=C**H_trans_**H_cis_), 6.27 (dd, J = 17.3, 10.5 Hz, 1H, **H**C=CH_trans_H_cis_), 5.99 (dd, J = 10.4, 1.2 Hz, 1H, HC=CH_trans_**H_cis_**).

^13^C [9] NMR (126 MHz, CDCl_3_) (δ): 164.46, 160.41 (dd, J = 257.6, 6.1 Hz), 152.20, 151.20 (ddd, J = 250.7, 10.7, 5.0 Hz), 146.20 (t, J = 10.6 Hz), 144.79 (td, J = 10.6, 4.0 Hz), 138.59 (dt, J = 250.4, 15.2 Hz), 135.27, 133.31, 128.28, 127.76, 119.32 (d, J = 266.1 Hz), 118.26, 116.24, 115.43, 111.13 (dd, J = 23.4, 3.5 Hz), 108.95 – 108.04 (m_apparent_), 107.59 (dd, J = 18.3, 5.6 Hz).

^19^F NMR (376 MHz, CDCl_3_) (δ): -61.66 (t, J = 26.2 Hz, 2F, O-C**F_2_**-Ar), -110.11 (td, J = 26.2, 11.1 Hz, 2F, Ar-**F**), -132.45 (dd, J = 20.6, 8.6 Hz, 2F, Ar-**F**), -163.14 (tt, J = 20.7, 5.9 Hz, 1F, Ar-**F**).

*4-acryloxy-2-fluoro-(3’,5’-difluoro-4’-(difluoro(3,4,5-trifluorophenoxy)methyl)-1,1’-biphenyl)* **(2)**

Yield: (Colourless crystals) 169 mg, 36%

R_F_ (50:50 Hexane:DCM): 0.51

^1^H NMR (501 MHz, CDCl_3_) (δ): 7.46 (t, J = 8.6 Hz, 1H, Ar-**H**), 7.20 (d_apparent_, J = 10.6 Hz, 2H, Ar-**H**), 7.12 – 7.06 (m, 2H, Ar-**H**)*, 6.99 (dd, J = 7.9, 5.8 Hz, 2H, Ar-**H**), 6.66 (dd, J = 17.3, 1.1 Hz, 1H, HC=C-**H_trans_**H_Cis_), 6.34 (dd, J = 17.3, 10.5 Hz, 1H, **H**C=CH_trans_H_cis_), 6.09 (dd, J = 10.4, 1.1 Hz, 1H HC=C-H_trans_**H_Cis_**).

*overlapping peaks

^13^C{^1^H} NMR (126 MHz, CDCl_3_) (δ): 164.01, 161.10 (dd, J = 257.7, 6.0 Hz), 159.35 (d, J = 250.9 Hz), 152.15 (dd, J = 11.0, 4.0 Hz), 150.16 (dd, J = 10.9, 5.2 Hz), 144.74 (td, J = 12.3, 3.9 Hz), 140.89 (t, J = 11.1 Hz), 138.12 (dt, J = 251.1, 15.2 Hz), 133.82, 130.65 (d, J = 3.8 Hz), 127.43, 123.33 (d, J = 12.7 Hz), 122.39, 120.27, 118.41 (d, J = 3.6 Hz), 118.15, 113.22 (dt, J = 23.8, 3.6 Hz), 110.87 (d, J = 25.7 Hz), 109.52 – 108.60 (m_apparent_), 107.62 (dd, J = 19.0, 6.0 Hz).

^13^C{^1^H}{^19^F} NMR (126 MHz, CDCl_3_) (δ): 163.87, 160.55, 159.94, 159.51, 158.47, 152.03, 144.60, 140.76, 133.68, 130.51, 127.30, 123.19, 120.14, 118.28, 118.04, 113.09, 110.74, 108.94, 107.48.

^19^F NMR (376 MHz, CDCl_3_) (δ): -61.78 (t, J = 26.4 Hz, 2F, O-C**F_2_**-Ar), -110.39 (td, J = 26.4, 11.1 Hz, 2F, Ar-**F**), -113.87 (t, J = 9.7 Hz, 1F, Ar-**F**), -132.43 (dd, J = 20.9, 8.7 Hz, 2F, Ar-**F**), -163.10 (tt, J = 20.8, 4.8 Hz, 1F, Ar-**F**).

*4-acryloxy-2,6-difluoro-(3’,5’-difluoro-4’-(difluoro(3,4,5-trifluorophenoxy)methyl)-1,1’-biphenyl)* ***(3)***

Yield: (Colourless crystals) 365 mg, 74%

R_F_ (50:50 Hexane:DCM): 0.55

^1^H NMR (501 MHz, CDCl_3_) (δ): 7.15 (d_apparent_, J = 10.1 Hz, 2H, Ar-**F**), 7.00 (dd, J = 7.9, 5.8 Hz, 2H, Ar-**F**), 6.96 – 6.90 (m_apparent_, 2H, Ar-**F**), 6.66 (dd, J = 17.3, 1.0 Hz, 1H, HC=C-**H_trans_**H_Cis_), 6.32 (dd, J = 17.3, 10.5 Hz, 1H, **H**C=CH_trans_H_cis_), 6.11 (dd, J = 10.5, 1.0 Hz, 1H, HC=C-H_trans_**H_Cis_**).

^13^C{^1^H} NMR (126 MHz, CDCl_3_) (δ): 163.58, 161.04 – 158.65 (m)^*^, 152.16 (ddd, J = 251.1, 10.7, 5.3 Hz), 151.87 (t, J = 14.4 Hz), 144.67 (td, J = 11.0, 1.7 Hz), 138.65 (dt, J = 250.4, 15.2 Hz), 134.51 (t, J = 11.5 Hz), 134.32, 127.11, 122.28, 120.16, 118.05, 115.25 – 114.59 (m_apparent_), 113.05 (t, J = 17.9 Hz), 110.23 – 109.37 (m_apparent_), 107.66 (dd, J = 18.7, 6.0 Hz), 106.72 (dd, J = 23.4, 6.9 Hz).

^*^Overlapping signals

^13^C{^1^H}{^19^F} NMR (126 MHz, CDCl_3_) (δ): 163.44, 159.75, 159.74, 159.72, 159.67, 151.74, 144.53, 139.59, 134.37, 134.18, 126.98, 120.03, 114.74, 112.91, 109.67, 107.52, 106.59.

^19^F NMR (400 MHz, CDCl_3_) (δ): -61.95 (t, J = 26.5 Hz, 2F, O-C**F_2_**-Ar), -110.59 (td, J = 26.6, 10.8 Hz, 2F, Ar-**F**), -112.06 (d, J = 9.3 Hz, 2F, Ar-**F**), -132.43 (dd, J = 20.7, 8.7 Hz, 2F, Ar-**F**), -163.06 (tt, J = 20.8, 6.0 Hz, 1F, Ar-**F**).

*4-acryloxy-2,3-difluoro-(3’,5’-difluoro-4’-(difluoro(3,4,5-trifluorophenoxy)methyl)-1,1’-biphenyl)* ***(4)***

Yield: (Colourless crystals) 278 mg, 56%

R_F_ (50:50 Hexane:DCM): 0.56

^1^H NMR (501 MHz, CDCl_3_) (δ): 7.25 – 7.17 (m, 3H, Ar-**H**)^*^, 7.14 – 7.08 (m_apparent_, 1H, Ar-**H**), 7.00 (dd, J = 7.9, 5.8 Hz, 2H, Ar-**H**), 6.70 (dd, J = 17.3, 1.0 Hz, 1H, HC=C-**H_trans_**H_Cis_), 6.38 (dd, J = 17.3, 10.5 Hz, 1H, **H**C=CH_trans_H_cis_), 6.14 (dd, J = 10.5, 1.0 Hz, 1H, HC=C-H_trans_**H_Cis_**).

^13^C{^1^H} NMR (126 MHz, CDCl_3_) (δ): 163.01, 160.15 (dd, J = 258.6, 5.8 Hz), 151.17 (ddd, J = 251.1, 10.7, 5.2 Hz), 148.81 (dd, J = 253.8, 12.0 Hz), 144.98 (dd, J = 253.8, 14.8 Hz), 144.66 (td, J = 11.4, 4.5 Hz), 140.21 – 139.79 (m), 138.66 (dt, J = 250.6, 14.9 Hz), 134.60, 126.55, 125.22 (d, J = 9.9 Hz), 123.75 (t_apparent_, J = 3.5 Hz), 122.29, 120.17, 119.29 (d, J = 4.0 Hz), 118.05, 113.27 (dd, J = 27.8, 3.3 Hz), 110.29 – 109.29 (m_apparent_), 107.64 (dd, J = 18.5, 5.6 Hz).

^13^C{^1^H}{^19^F} NMR (126 MHz, CDCl_3_) (δ): 162.88, 160.02, 160.00, 144.53, 142.84, 139.94, 139.77, 139.55, 137.47, 134.46, 126.42, 125.08, 123.61, 120.03, 119.15, 113.13, 109.53, 107.50.

^19^F NMR (376 MHz, CDCl_3_) (δ): -61.86 (t, *J* = 26.4 Hz, 2F, O-C**F_2_**-Ar ), -109.79 (td, *J* = 26.4, 10.8 Hz, 2F, Ar-**F**), -132.37 (dd, *J* = 20.7, 8.7 Hz, 2F, Ar-**F**), -138.86 (dd, *J* = 20.2, 7.4 Hz, 1F, Ar-**F**), -148.57 (dd, *J* = 20.3, 6.7 Hz, 1F, Ar-**F**), -162.99 (tt, *J* = 21.0, 6.0 Hz, 1F, Ar-**F**).

## NMR Spectra

**Figure S1** - NMR spectra (In descending order:^1^H, ^13^C{^1^H}, and ^19^F, respectively), for **(1)**.

**Figure S2** - NMR spectra (In descending order: ^1^H, ^13^C{^1^H}, ^13^C{^1^H}{^19^F}, and ^19^F, respectively) **(2)**.

**Figure S3** - NMR spectra (In descending order: ^1^H, ^13^C{^1^H}, ^13^C{^1^H}{^19^F}, and ^19^F, respectively) **(3)**.

**Figure S4** - NMR spectra (In descending order: ^1^H, ^13^C{^1^H}, ^13^C{^1^H}{^19^F}, and ^19^F, respectively) **(4)**.

## Characterisation of Monomers


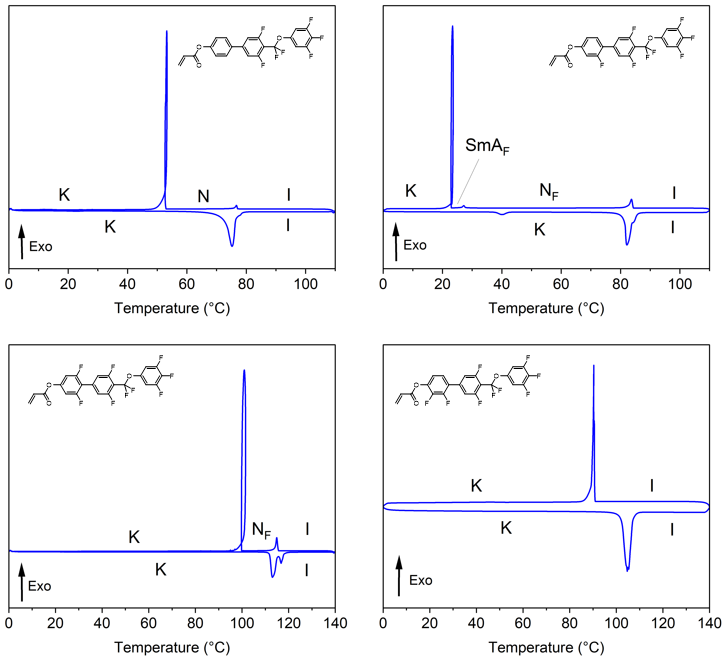


**Figure S5** – Example DSC thermograms for the synthesise monomers.


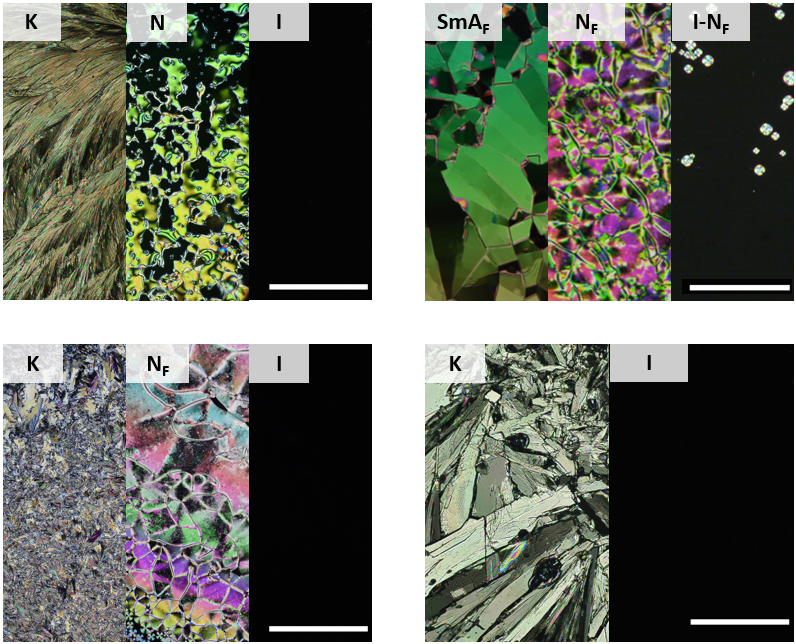


**Figure S6** – POM micrographs to show the phase behaviour for **1** (top left), **2** (top right), **3** (bottom left) and **4** (bottom right). In all cases the scale bar represents 500 µm.


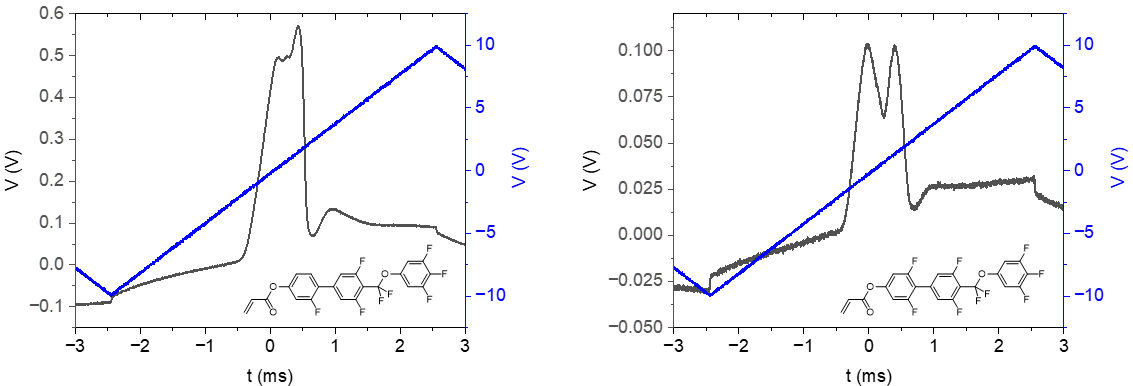


**Figure S7** – Current response traces measured for 2 and 3, confirming the assignment of the N_F_ phase. In both cases measurements were taken at 100 Hz, at temperatures of 70 °C and 100 °C for monomers **2** and **3** respectively.

## Polymer Stabilisation Studies

### Procedure

The appropriate quantities of **F7**, **RM82**, **2**, and **MBF** were added to DCM (2 mL), and the mixture stirred for 5 minutes. The solvent was then removed at elevated temperature to yield the desired mixture. The resulting mixture was sparged with nitrogen for 5 minutes before being filled into the appropriate sample container (e.g. liquid crystal cell). The mixtures were then subject to irradiation at 365 nm (2.5 W cm^-2^) for 20 minutes whilst at room temperature, to yield the polymer stabilised mixture.

### Characterisation


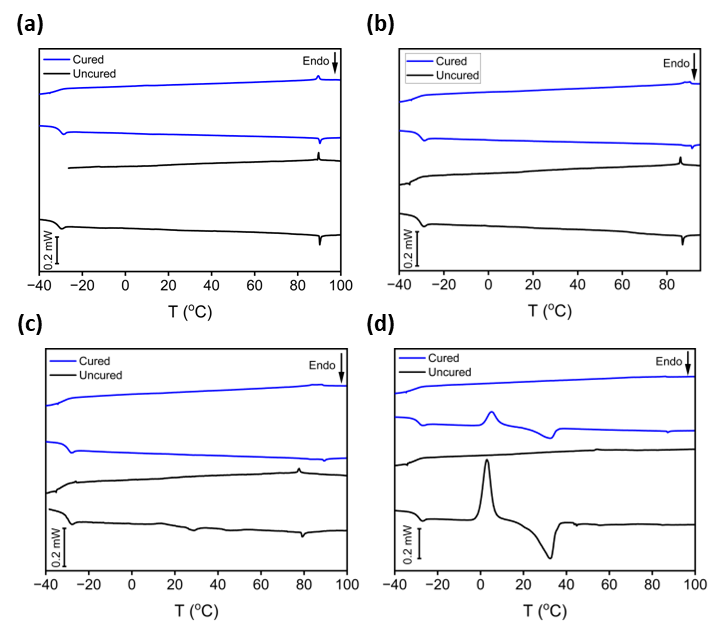


**Figure S8** – DSC thermograms for the polymer stabilisation experiments on mixtures PS1 (**(a)**), PS2 (**(b)**), PS3 (**(c)**) and PS4 (**(d)**). In all cases, the blue thermogram represents the cured mixture, and the black thermogram represents the uncured mixture.


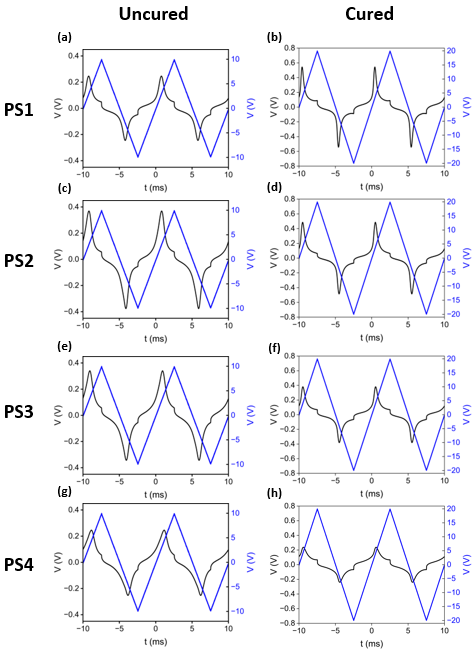


**Figure S9** – Current response traces for the polymer stabilisation mixtures. Panels **(a), (c), (e)** and **(g)** were acquired for uncured samples of **PS1, PS2, PS3** and **PS4** respectively. Panels **(b), (d), (f)** and **(h)** were obtained for cured samples of **PS1, PS2, PS3** and **PS4** respectively.


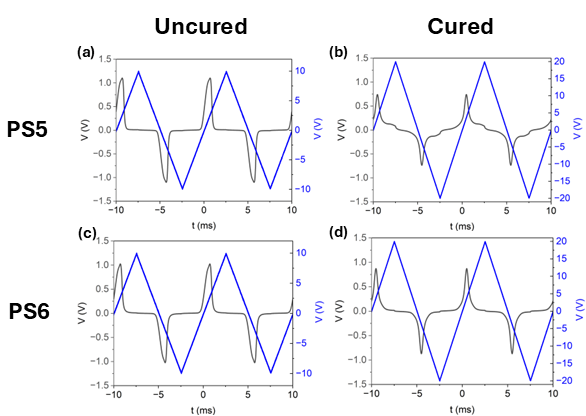


**Figure S10** – Current response traces for the polymer stabilisation mixtures. Panels **(a)** and **(c)** were acquired for uncured samples of **PS5** and **PS6** respectively. Panels **(b)** and **(d)** were obtained for cured samples of **PS5** and **PS6** respectively.

### Mixture Degradation at Elevated Temperatures

**Figure S11** – a) DSC studies showing the stability of the T_NF_ transition when cycled up to 120 °C for the PS3 mixture, and b) DSC studies showing the degradation of material, due to isomerisation of DIO, resulting in a reduction in T_NF_ when repeatedly cycled to increasingly higher temperatures, again for the PS3 mixture.

## Three Dimensional Electrostatic Potential Computations

**
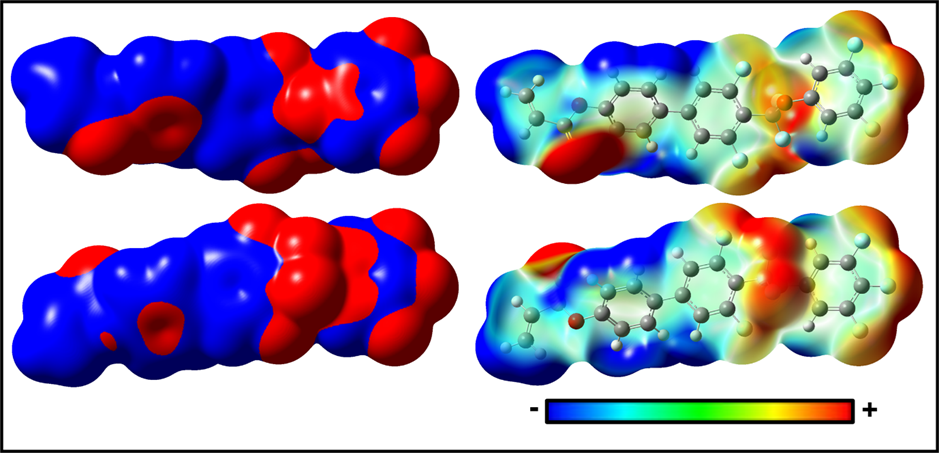
**

**Figure S12** – 3D ESP plots for **1** calculated at an electron density of 0.0004 using the B3LYP-GD3BJ/cc-pVTZ level of theory. Top and bottom show the different sides of the molecule while molecules on the left at set so that all negatively charged regions are shaded red and all positively charged regions are blue.


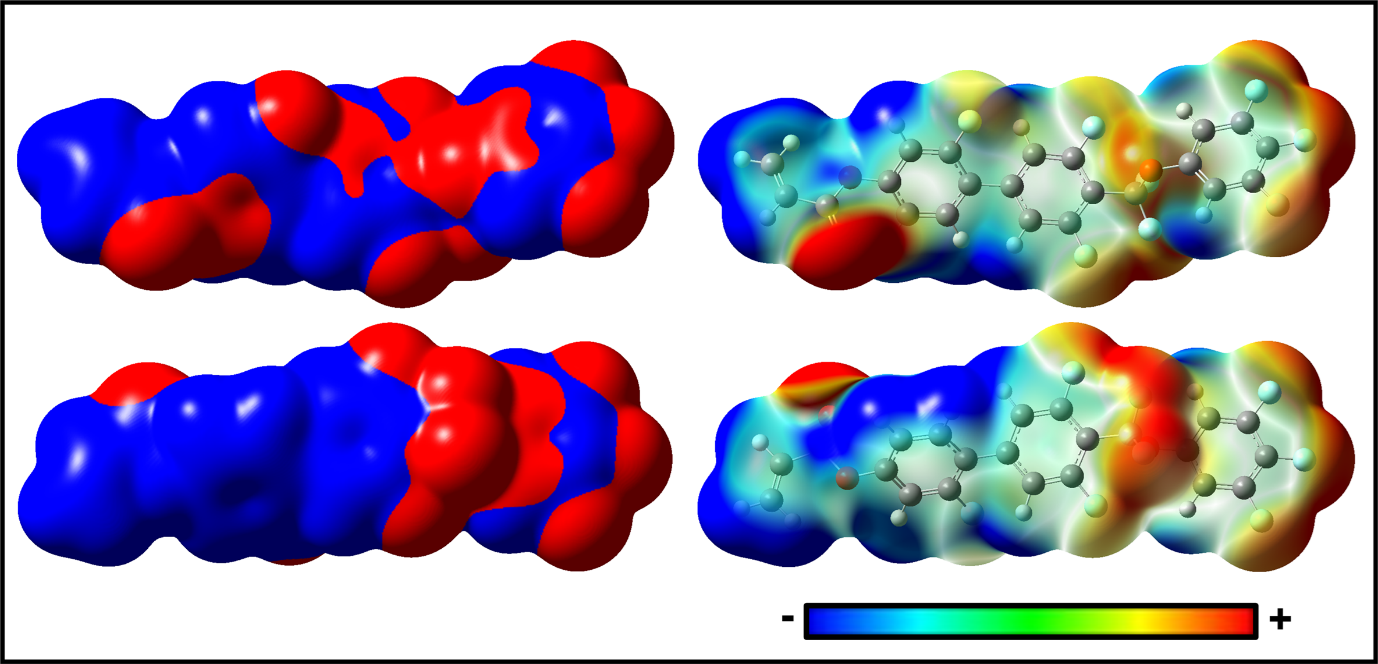


**Figure S13** – 3D ESP plots for **2** calculated at an electron density of 0.0004 using the B3LYP-GD3BJ/cc-pVTZ level of theory. Top and bottom show the different sides of the molecule while molecules on the left at set so that all negatively charged regions are shaded red and all positively charged regions are blue.


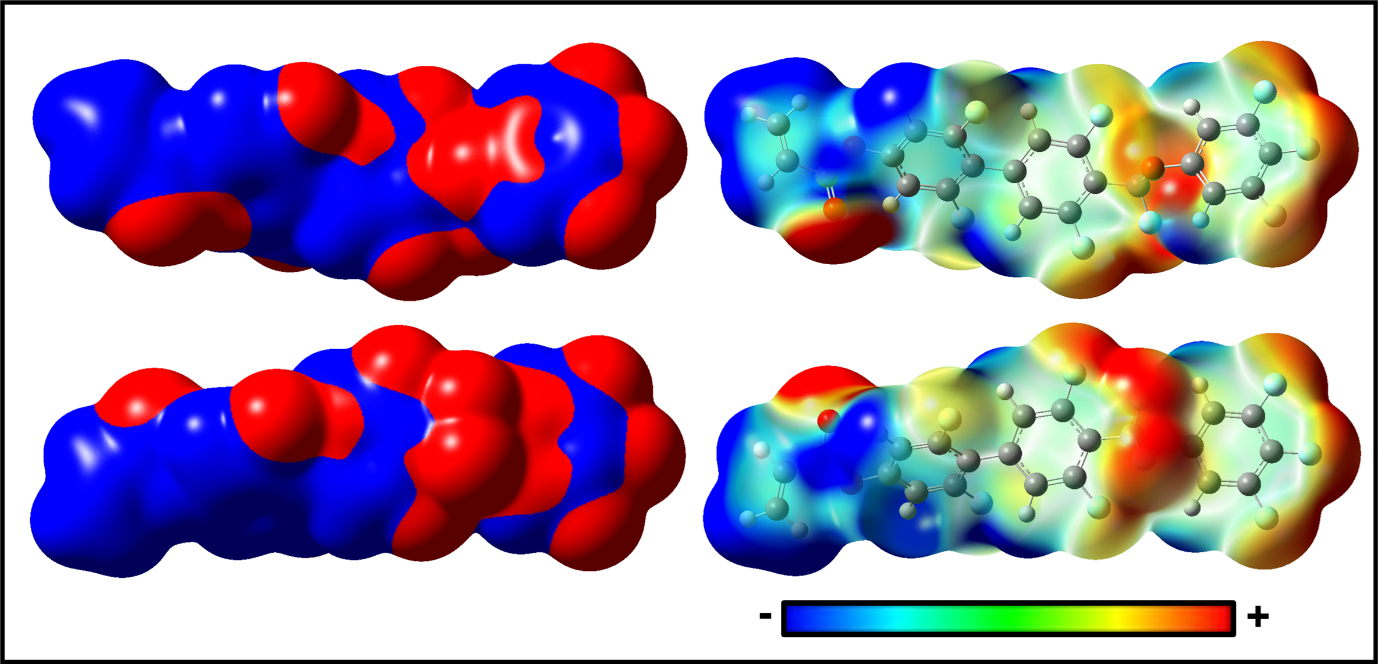


**Figure S14** – 3D ESP plots for **3** calculated at an electron density of 0.0004 using the B3LYP-GD3BJ/cc-pVTZ level of theory. Top and bottom show the different sides of the molecule while molecules on the left at set so that all negatively charged regions are shaded red and all positively charged regions are blue.


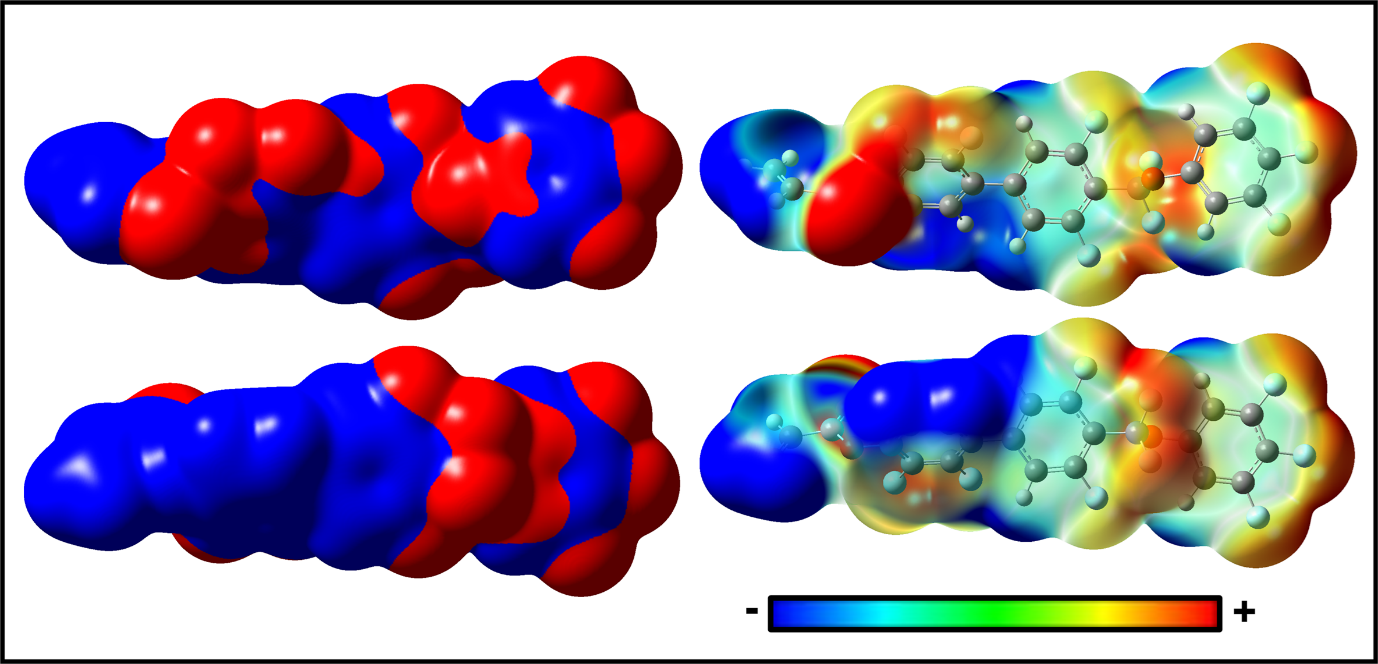


**Figure S15** – 3D ESP plots for **4** calculated at an electron density of 0.0004 using the B3LYP-GD3BJ/cc-pVTZ level of theory. Top and bottom show the different sides of the molecule while molecules on the left at set so that all negatively charged regions are shaded red and all positively charged regions are blue.

## References

1. Frisch, M. J.; Trucks, G. W.; Schlegel, H. B.; Scuseria, G. E.; Robb, M. A.; Cheeseman, J. R.; Scalmani, G.; Barone, V.; Petersson, G. A.; Nakatsuji, H.; Li, X.; Caricato, M.; Marenich, A. V.; Bloino, J.; Janesko, B. G.; Gomperts, R.; Mennucci, B.; Hratchian, H. P.; Ortiz, J. V.; Izmaylov, A. F.; Sonnenberg, J. L.; Williams; Ding, F.; Lipparini, F.; Egidi, F.; Goings, J.; Peng, B.; Petrone, A.; Henderson, T.; Ranasinghe, D.; Zakrzewski, V. G.; Gao, J.; Rega, N.; Zheng, G.; Liang, W.; Hada, M.; Ehara, M.; Toyota, K.; Fukuda, R.; Hasegawa, J.; Ishida, M.; Nakajima, T.; Honda, Y.; Kitao, O.; Nakai, H.; Vreven, T.; Throssell, K.; Montgomery Jr., J. A.; Peralta, J. E.; Ogliaro, F.; Bearpark, M. J.; Heyd, J. J.; Brothers, E. N.; Kudin, K. N.; Staroverov, V. N.; Keith, T. A.; Kobayashi, R.; Normand, J.; Raghavachari, K.; Rendell, A. P.; Burant, J. C.; Iyengar, S. S.; Tomasi, J.; Cossi, M.; Millam, J. M.; Klene, M.; Adamo, C.; Cammi, R.; Ochterski, J. W.; Martin, R. L.; Morokuma, K.; Farkas, O.; Foresman, J. B.; Fox, D. J. *Gaussian 16 Rev. C.01*, Wallingford, CT, **2016**.

2. Lee, C.; Weitao, Y.; Parr, R. G., *Phys. Rev. B* **1988,** *37* (2), 785.

3. Becke, A. D., *J. Chem. Phys.* **1993,** *98* (7), 5648-5652. DOI 10.1063/1.464913.

4. Dunning, T. H., Jr., *J. Chem. Phys.* **1989,** *90* (2), 1007-1023. DOI 10.1063/1.456153.

5. Grimme, S.; Ehrlich, S.; Goerigk, L., *J. Comput. Chem.* **2011,** *32* (7), 1456-1465. DOI https://doi.org/10.1002/jcc.21759.

6. Martinot-Lagarde, P., *J. Phys. Lett.* **1977,** *38* (1), 17-19.

7. Miyasato, K.; Abe, S.; Takezoe, H.; Fukuda, A.; Kuze, E., *Jpn. J. Appl. Phys.* **1983,** *22* (10A), L661. DOI 10.1143/JJAP.22.L661.

8. Gibb, C. J.; Hobbs, J.; Nikolova, D. I.; Raistrick, T.; Berrow, S. R.; Mertelj, A.; Osterman, N.; Sebastián, N.; Gleeson, H. F.; Mandle, R. J., *Nat. Commun.* **2024,** *15* (1), 5845. DOI 10.1038/s41467-024-50230-2.

9. Jordan Hobbs, C. J. G., Richard J. Mandle *ArXiv* **2025**. DOI https://doi.org/10.48550/arXiv.2502.08551.

10. Karcz, J.; Herman, J.; Rychłowicz, N.; Kula, P.; Górecka, E.; Szydlowska, J.; Majewski, P. W.; Pociecha, D., *Science* **2024,** *384* (6700), 1096-1099. DOI doi:10.1126/science.adn6812.
